# Increased risk of cancer in patients with type 2 diabetes mellitus: A retrospective cohort study in China

**DOI:** 10.1186/1471-2458-12-567

**Published:** 2012-07-28

**Authors:** Pian-Hong Zhang, Zhong-Wen Chen, Duo Lv, Yu-Yang Xu, Wei-Ling Gu, Xu-Hui Zhang, Yuan-Luo Le, Hong-hong Zhu, Yi-Min Zhu

**Affiliations:** 1 Second Affiliated Hospital, Zhejiang University School of Medicine, Hangzhou, 310009, People's Republic of China; 2 Jiaxing Center for Disease Control and Prevention, Jiaxing, 314050, People's Republic of China; 3 Department of Epidemiology and Biostatistics, Zhejiang University School of Medicine, 388 Yu-Hang-Tang Road, Hangzhou, 310058, Zhejiang, People's Republic of China; 4 Hangzhou Center for Disease Control and Prevention, Hangzhou, 310021, People's Republic of China; 5 Department of Public Health, College of Health and Human Services, Western Kentucky University, Bowling Green, KY, 42101, USA

## Abstract

**Background:**

Previous studies indicated that type 2 diabetes mellitus (T2DM) might be associated with the risk of cancer. The aim of this study was to investigate the association between T2DM and the risk of developing common cancers in a Chinese population.

**Methods:**

A population-based retrospective cohort study was carried out in the Nan-Hu district of Jiaxing city, Zhejiang province, China. The incidence of cancer cases among type 2 diabetic patients were identified through record-linkage of the Diabetic Surveillance and Registry Database with the Cancer Database from January 2002 to June 2008. The standardized incidence ratio (SIR) and 95% confidence interval (CI) were estimated for the risk of cancer among the patients with type 2 diabetes.

**Results:**

The overall incidence of cancer was 1083.6 per 10^5^ subjects in male T2DM patients and 870.2 per 10^5^ in females. Increased risk of developing cancer was found in both male and female T2DM patients with an SIR of 1.331 (95% CI = 1.143-1.518) and 1.737 (1.478-1.997), respectively. As for cancer subtypes, both male and female T2DM patients had a significantly increased risk of pancreatic cancer with the SIRs of 2.973 (1.73-4.21) and 2.687 (1.445-3.928), respectively. Elevated risk of liver and kidney cancers was only found in male T2DM patients with SIRs of 1.538 (1.005-2.072) and 4.091 (1.418-6.764), respectively. Increased risks of developing breast cancer [2.209 (1.487-2.93)] and leukemia SIR: [4.167 (1.584- 6.749) ] were found in female patients.

**Conclusions:**

These findings indicated that patients with T2DM have an increased risk of developing cancer. Additional cancer screening should be employed in the management of patients with T2DM.

## Background

Type 2 diabetes mellitus (T2DM) is prevalent all over the world. The incidence of diabetes for all age groups worldwide was estimated to be 2.8% in 2000 and is projected to be 4.4% in 2030 [1]. It is estimated that the total number of people with diabetes will increase from 171 million in 2000 to 366 million in 2030 [1]. In China, the latest statistical data indicates that the prevalence of diabetes and pre-diabetes in adults older than 20 years is 9.7% and 15.5%, respectively. This means that there are 92 million diabetic patients and 148 million pre-diabetic patients [2]. T2DM is associated with increased risks of developing coronary heart disease and stroke [3,4] and has been becoming a growing major public health problem worldwide.

A link between diabetes and cancer was first proposed in 1934 and has been investigated extensively [5]. Epidemiologic evidence has suggested that diabetes increases the risk of developing several cancers [6] such as pancreatic [7] and liver cancer [8,9], colorectal cancer [10-12], breast cancer [13], bladder cancer [14], kidney cancer [15] and others. A reduced risk of prostate cancer was found in male diabetes patients [16]. However, previously reported findings have not all been consistent. For example, no significant association between diabetes and prostate cancer was found in nine case–control studies [17]. The inconsistency in findings from previous epidemiological studies might be due to the use of different study design (inclusion criteria), incomplete characterization of subjects, and confounding variables such as obesity, duration of diabetes and treatment [17]. Diagnosis of diabetes in some previous studies was based on self-report and did not distinguish between T2DM and T1DM [18,19]. The majority of the published studies have been carried out in Western populations. No cohort study has been reported on diabetes and the risk of developing cancer in the Chinese population. The generalization of findings from Western populations to Chinese may be problematic due to their difference in genetic susceptibility, environmental exposure, and life style etc. Woodward et al. [20] found that the risk of cardiovascular disease and cancer from diabetes among the Asian population might be different from that among the Western population. Therefore, study on a relatively leaner population in China (mean body mass index [BMI] of 23.6) will be useful in further strengthening evidence on the association between diabetes and cancer. One of our recent studies [21] showed that T2DM was significantly associated with an increased risk of developing colon cancer but not rectal cancer. Considering the high prevalence of T2DM all over the world, even a small increase in the cancer risk might have a significant public health impact at the population level.

In the present study, we investigated the associations of T2DM with other common cancers in Chinese by means of a population-based risk analysis.

## Methods

### Study participants

The location and subjects involved in this study have been described previously [21]. Briefly, the location was Nan-Hu district of Jiaxing city, Zhejiang province, China, which is situated in southeastern China. This region has one of the highest incidences of colorectal cancer in China. The total population in 2002 was 454,155. In this district, cancer and diabetes surveillance systems have been set up since 1990 and 2002, respectively. Once a specific patient with cancer or diabetes based on the 1999 WHO criteria [22] was diagnosed by the certificated physicians in the regional hospitals, the patient information was reported to the regional Centers of Disease Control and Prevention (CDC), and then the data were verified and recorded in the Cancer/ Diabetics Surveillance and Registry Database (CSRD and DSRD). The missing reporting rate was less than 5%. Both the surveillance systems for cancer and diabetes were parts of National Cancer and Diabetic Surveillance Systems in China.

Subjects in this study were the clinically reported patients with T2DM (ICD-10 code: E11) in Nan-Hu district from 1 January 2002 to 30 June 2008. The incidence of cancer in all of the subjects was identified through record-linkage of DSRD with CSRD. The common cancers in this study identified by ICD-10 codes included lung and bronchus (C33-C34), stomach (C16), liver (C22), kidney (C64), esophagus (C15), pancreas (C25), uterine cervix (C53), uterine corpus (C54), ovary (C56), breast (C50), leukemia (C91-C95), prostate (C61) and urinary bladder (C67).

This study was approved by the Institutional Review Board of Zhejiang University School of Public Health.

### Statistical analysis

Person-years of follow-up for each T2DM patient were calculated from the date that T2DM was first diagnosed to the date on which common cancer was diagnosed, or the June 30, 2010, whichever occurred first. Overall, gender- and sub-site incidence rates for specific cancers were calculated by the number of incidence cases divided by the number of observed person-years. Ninety-five percent confident intervals (95% CI) for incidence rates were calculated based on the Poisson distribution. The age-standardized rate (ASR [W]) was calculated using the World Standard Population. The standardized incidence ratio (SIR) and its 95% CI were used to estimate the risk of common cancer in subjects with T2DM. For calculation of SIR, the number of expected cancer cases in T2DM patients was calculated first based on the age- and gender-specific cancer incidence rates in the Nan-Hu general population as the standardized incidence rate during the same period, and SIR was calculated as the ratio of the number of observed cancer cases to the number of expected cancer cases using following formula:

(1) $$SIR=\frac{\text{the number of observed cancer cases }}{\text{the number of expected cancer cases}}$$

The 95% CI of SIR was calculated using the following formula:

(2) $$SIR\pm 1.96\times S_{e}$$

where Se=SIRnumber of observed cases

The databases were managed using Microsoft Excel software and all the statistical calculations were peroformed using SAS v9.2 (SAS Institute Inc., Cary, NC, USA).

## Results

### Basic characteristics of patients with T2DM in the cohort

The characteristics of T2DM patients in this study were described previously [21]. From 1 January 2002 to 30 June 2008, a total of 7950 T2DM patients were reported through the Diabetics Surveillance and Registry Database system in Nan-Hu district, Jiaxing City. Among 7950 type 2 diabetic patients, 3795 (47.7%) were men and 4155 (52.3%) were women. The mean age at the diagnosis of diabetes was 61.1 years, the mean BMI was 23.6 kg/m^2^, and 792 (9.98%) diabetic patients had family histories of diabetes. The total observed time was 37669.9 person-years. During 1 January 2002 to 30 June 2010, 366 cases of cancers in 7950 T2DM patients were identified through record-linkage of DSRD with CSRD. Of these cancer cases, 194 were men and 172 were women.

The crude incidence rate (CIR) and ASR (W) of specific cancers in male and female T2DM patients are shown in Table 1. The overall CIR of cancers in men and women was 1083.6 and 870.2 per 10^5^ person-years, respectively, and the ASR (W) in men and women was 270.7 and 250.4 per 10^5^ person-years, respectively.

**Table 1:** Incidence rates* of cancers in patients with type 2 diabetes

	**Male (N = 3795)**			**Female (N = 4155)**		
	**n**	**CIR**	**ASR**	**n**	**CIR**	**ASR**
All cancers	194	1083.6	270.7	172	870.2	250.4
lung cancer	41	229.0	53.7	25	126.5	30.8
kidney cancer	9	50.3	21.0	6	30.4	6.5
stomach cancer	25	139.6	38.0	16	80.9	17.6
liver cancer	32	178.7	949.9	16	80.9	112.7
esophagus cancer	6	33.5	10.1	3	15.2	3.9
pancreas cancer	22	122.9	25.5	18	91.1	17.5
leukemia cancer	7	39.1	21.6	10	50.6	20.8
prostate cancer	12	67.0	13.3			
urinary bladder cancer	4	22.3	3.3			
uterine cervix cancer				4	20.2	5.5
uterine corpus cancer				5	25.3	6.9
ovary cancer				5	25.3	6.1
breast cancer				36	182.1	68.5

*CIR: crude incidence rate (per 100000 person years); ASR: Age-standardized rate (per 100000 person years) calculated using World Standard Population data.

### SIRs of common cancers in patients with T2DM by gender and subtype of cancer

SIRs of common cancers in T2DM patients by gender and subtype of cancer are shown in Table 2. Increased risks of overall cancer in T2DM patients were found in men and women with SIRs of 1.331 (95% CI = 1.143-1.518) and 1.737 (95% CI = 1.478-1.997), respectively. For subtypes of cancer, men and women T2DM patients had significantly increased risks of pancreatic cancer with SIRs of 2.973 (95% CI = 1.731-4.215) and 2.687 (1.445-3.928), respectively. Elevated risks of liver and kidney cancers were found only in male T2DM patients with SIRs of 1.538 (1.005-2.072) and 4.091 (1.418-6.764), respectively. Women patients had significantly increased risk of breast (2.209 [1.487-2.93]) and leukemia (4.167 [1.584-6.749]) cancers. In this study, no significant link was found between T2DM and increased risk of cancer of the lung, stomach, esophagus, bladder, prostate, uterine cervix, uterine corpus or ovary.

**Table 2:** SIRs of cancers in patients with type 2 diabetes by gender and subtype of disease

	**Male (N = 3795)**					**Female (N = 4155)**				
	**Cases**		**SIR†**	95% CI*		**Cases**		**SIR**	**95% CI**	
	**Observed**	**Expected**		**Low**	**Upper**	**Observed**	**Expected**		**Low**	**Upper**
All cancers	194	145.8	1.331	1.143	1.518	172	99.0	1.737	1.478	1.997
Lung & bronchus	41	48.4	0.847	0.588	1.106	25	18.3	1.366	0.831	1.902
Kidney	9	2.2	4.091	1.418	6.764	6	1.5	4.00	0.799	7.201
Stomach	25	20.5	1.22	0.741	1.698	16	10.3	1.553	.792	2.315
Liver	32	20.8	1.538	1.005	2.072	16	11.0	1.455	0.742	2.167
Esophagus	6	12.6	0.476	0.095	0.857	3	3.5	0.857	0.0	1.827
Pancreas	22	7.4	2.973	1.731	4.215	18	6.7	2.687	1.445	3.928
Uterine cervix						4	2.4	1.667	0.033	3.30
Uterine corpus						5	2.8	1.786	0.22.0	3.351
Ovary						5	3.2	1.563	0.19.3	2.932
Breast						36	16.3	2.209	1.487	2.93
Leukemia	7	2.1	3.333	0.864	5.803	10	2.4	4.167	1.584	6.749
Prostate	12	6.4	1.875	0.814	2.936					
Urinary bladder	4	5.7	0.702	0.014	1.389	1	1.5	0.667	0.0	1.973

*95% CI, 95% confidence interval.

†, Number of expected cancer incidence cases was calculated according to the age- and gender-incidence level of the general population in the surveillance region and the observed person-years in type 2 diabetes.

## Discussion

In this study, we investigated the association between T2DM and the risk of developing cancer by means of analysis of population-based registry data in China. Our findings indicated that both men and women patients with T2DM had an increased risk of pancreatic cancer; men had an increased risk of liver and kidney cancer and women had an increased risk of breast cancer and leukemia. We have previously reported the increased risk of developing colorectal cancer in T2DM patients [21]. In terms of anatomic sub-site, T2DM was significantly associated with an increased risk of colon cancer but not rectal cancer. Taken together, these findings indicated that Chinese patients with T2DM had an increased risk of developing several types of cancer.

The association between T2DM and the risk of developing common cancer has been extensively investigated. Wideroff et al. [6] found that diabetic patients increased risk of developing cancer by only 10% (SIR = 1.1), in an analysis involving all types of cancers in Denmark. However, no significant association was found between T2DM and an increased incidence of cancer (RR = 0.99, 95% CI = 0.90-1.09) in a UK study [23]. In this study, we found an increased risk of cancer in T2DM in Chinese population. Asia populations including Chinese have relatively leaner and lower body mass index. The mean BMI of subjects in this study was 23.6. Inconsistency results among studies in difference countries indicate there may be ethnic difference on biologic effect of T2DM. In a population-based retrospective cohort study in Japan, Oba et al. found that diabetic patients had an increased risk of cancer mortality [19]. Woodward et al. [20] also found that the risk of cardiovascular disease and cancer from diabetes among the Asian population might be different from that among the western population. A meta-analysis of 23 studies involving preexisting diabetes mellitus, carried out by Barone et al. [24] indicated that diabetes was associated with increased mortality with an HR of 1.41 (1.28-1.55) as compared with normoglycemic individuals across all cancer types.

The cancer risks in diabetes patients varies depending on the sub-sites of specific cancers. Our findings and results from previous studies indicate that the strongest association between T2DM and cancer risk is with pancreatic cancer [17]. As compared with the general population, we found that male and female T2DM patients were 2.97 and 2.68 times more likely to develop pancreatic cancer, respectively. This finding was in agreement with previous studies [18,25]. The pooled risk of developing pancreatic cancer in T2DM was 1.73 (1.59-1.88) [26]. However, this association may be involved in a mix of patients with pre-existing diabetes and new-onset diabetes. New-onset diabetes is common in lean patients aged over 45 years.

Our findings also indicated an association between T2DM and liver cancer in the Chinese men population. The results from a study by Oba et al. indicated that T2DM increased the risk of liver cancer with an adjusted relative risk (RR) of 2.93 (1.40-6.14) [27]. Although the exact mechanisms underlying this association are still unclear, the following factors are attributable to an increased liver cancer risk in diabetic patients: the mitogenic action of insulin; liver inflammation; hepatocyte damage and repair; diabetes-related diseases of liver cancer, such as steatosis and cirrhosis; and nonalcoholic fatty liver disease [28].

Nine cohort studies had examined the association between T2DM and kidney cancer, the pooled risk of kidney cancer was 1.42 (95% CI = 1.06-1.91) [15]. About 3 times of kidney cancer risk was found in present study. Diabetes might increase the risk of kidney cancer by hyperinsulinemia and insulin resistance, higher IGF-1 in serum and hypertension. Chronic kidney diseases are common in diabetic patients.

T2DM was also found to be associated with an increased risk of colon, bladder, breast and endometrial cancers [10,12,15,29-31]. However, in contrast to previous studies, we did not find a significant association between bladder and endometrial cancer. This finding might be attributable to population differences, inadequate sample size and short observational periods. Most previous studies reported a reduced risk of prostate cancer in diabetic patients [32-35]. Consistent with nine previous case–control studies, no significant association was found with prostate cancer in the present study. There might be site-specific mechanisms involved in carcinogenesis in different organs.

Several mechanisms have been proposed to explain the relationship between T2DM and cancer risk. Diabetes is one of the most common endocrine disorders today, which is caused by both environmental and genetic factors. T2DM and common cancers share many risk factors, including age, obesity, higher intake of saturated fats and refined carbohydrates, sedentary lifestyle, tobacco smoking and some psychology factors [28]. Shared risk factors could partly explain the clustering of T2DM and cancers. On the other hand, T2DM develops as a consequence of relative insulin insufficiency and is characterized by hyperinsulinemia and insulin resistance for compensation. Insulin is a well-recognized growth factor. Hyperinsulinemia promotes the proliferation of colon cancer cells in vitro and colonic tumors in experimental animals [36]. Therefore, hyperinsulinemia might promote carcinogenesis directly [37]. Through the interaction with insulin-like growth factor axis protein 1(IFG-1) and insulin-like growth factor binding proteins, hyperinsulinemia also might increase the level of IGF-1. IGF-1 is a potential mitogen and inhibitor of apoptosis [38,39] and also can promote tumorigenesis in various cancer model systems. The plasma or serum level of IGF-1 was found to be associated with an increased risk of cancer [40,41]. Recently, Jin et al. [42] speculated that the compensation for glucagon-like peptide-1 by intestinal endocrine L cells activated the Wnt signaling pathway and cross-talk between Wnt and insulin signaling pathways. Therefore, shared environmental factors and insulin resistance support the association between T2DM and risk of developing cancer.

The present study had several strengths. It was a population-based cohort study with large sample size of 7950 T2DM patients, having a median follow-up length of more than 8 years. T2DM was diagnosed by clinicians, and patient data were recorded in the regional CDC, detected and verified in the CSRD and DSRD. The surveillance systems for both diabetes and cancer are components of the National Cancer and Diabetic Surveillance Systems in China. The representativeness of patients in our study was relatively high.

Nonetheless, our study had some limitations. Although the study provided large number of diabetes patients, number of some subtype cancer cases is relatively small. A small subtype cancer could lead to a low statistical power to detect significant association between T2DM and some subtype cancers such as cancers of the bladder, endometrium and lung. In addition, although age and gender were controlled in calculation of SIR, this study lacked the information of other confounding factors such as body mass index (BMI), obesity status or type of diabetes treatment. BMI was reported to be associated with increased the risk of both of T2DM and cancers [43]. The antidiabetic drug metformin was found to be associated with reduced the risk of cancers [44,45], so reduced association estimation might existed in this study. Diabetes is an under-diagnosed disease and some T2DM patients were not be diagnosed in this study and might be included as population controls. This would lead to diluted association.

In summary, the present study provides valuable evidence that T2DM increases the risk of developing cancer in Chinese population. This association was more evident for pancreas, liver, kidney and breast cancers and leukemia. Considering the high prevalence of T2DM in the population, even a small increase in cancer risk might have substantial consequences at the population level. Therefore, further examinations, such as frequent colonoscopy for colorectal cancer and other screening test for other cancers, should be employed in the management of T2DM patients.

## Conclusions

Findings of this study indicate that T2DM is associated with an increased risk of cancer. Additional cancer screening should be employed in the management of patients with T2DM.

## Abbreviations

T2DM: Type 2 diabetes mellitus; SIR: Standardized incidence ratio; CDC: Centers of Disease Control and Prevention; CSRD: Cancer Surveillance and Registry Database; DSRD: Diabetics Surveillance and Registry; ASR (W): Age- standardized rate using World Standard Population; CIR: Crude incidence rate; IFG-1: Insulin- insulin like growth factor 1; HR: Hazard ratio.

## Competing of interests

None of the authors have conflict of interests to declare.

## Authors’ contributions

PHZ participated in the epidemiological investigation and drafted the manuscript; CHZ was responsible for the data collection; XYY, XHZ, YLL and WLG participated in the epidemiological investigation; DL carried out data analysis; YMZ participated in the overall design, study coordination, data analysis and finalized the draft of the manuscript. HHZ interpreted the results and refined the manuscript. All of the authors read and approved the final manuscript.

## Pre-publication history

The pre-publication history for this paper can be accessed here:

http://www.biomedcentral.com/1471-2458/12/567/prepub
